# Epidemiological impact and cost‐effectiveness analysis of PrEP provision expansion among MSM in the Netherlands

**DOI:** 10.1002/jia2.26516

**Published:** 2025-06-03

**Authors:** Haoyi Wang, Stephanie Popping, David van de Vijver, Kai J. Jonas

**Affiliations:** ^1^ Department of Work and Social Psychology Maastricht University Maastricht The Netherlands; ^2^ Viroscience Department Erasmus Medical Centre Rotterdam The Netherlands; ^3^ Department of Medical Microbiology and Infection Prevention Amsterdam University Medical Center, University of Amsterdam Rotterdam The Netherlands; ^4^ Cener for Infection and Molecular Medicine (CIMM) Amsterdam University Medical Center Amsterdam The Netherlands; ^5^ Amsterdam Institute for Immunology and Infectious Diseases Amsterdam The Netherlands

**Keywords:** budget impact analysis, cost‐effectiveness analysis, Europe, HIV, mathematical modelling, men who have sex with men, pre‐exposure prophylaxis

## Abstract

**Introduction:**

Several European countries show potential for pre‐exposure prophylaxis (PrEP) provision expansion, with many men who have sex with men (MSM) on waiting lists. In the Netherlands, approximately 15,000 PrEP‐eligible/intending MSM are awaiting PrEP access. We modelled the epidemiological and economic impact of extending PrEP provision considering several PrEP provision routes (National PrEP Programme and alternative PrEP providers).

**Methods:**

We calibrated our HIV transmission model among the Dutch MSM epidemic. PrEP was expanded from 2022 onwards, covering an additional 3000 MSM on the waiting list and in addition one‐third (5000), two‐thirds (10,000), and all (15,000) PrEP‐eligible/intending MSM by 2024, compared to a non‐expansion scenario. The epidemiological impact was projected by 2030. Costs were calculated from a third‐party payer's perspective over 40 years with Dutch‐specific quality‐adjusted life years (QALY). Additionally, a budget impact analysis was performed over 5 years.

**Results:**

Covering the 3000 waiting‐list MSM, one‐third, two‐thirds and all PrEP eligible/intending MSM by 2024 will avert 17 (5.7%), 46 (15.2%), 88 (29.1%) and 115 (37.9%) cumulative new HIV acquisitions compared to the base‐case scenario. Consequently, 4, 2, 0 and 0 new HIV acquisitions will result by 2030, respectively. The epidemiological impact of PrEP expansion is sensitive to the users’ PrEP adherence, but overall minimal by PrEP targeting strategies, given the strongly declining epidemic. Increasing the National PrEP Programme's capacity incurred more costs to the payer (short‐term budget impact ranging from €2.25 to €45.29 million). PrEP expansion can be cost‐saving when all PrEP‐eligible/intending MSM are covered and fully provided by alternative PrEP providers, with an incremental cost‐effectiveness ratio of −€2160/QALY over 40 years. This scenario dominated over all other scenarios. Our cost‐effectiveness analysis is most sensitive to the individual co‐payment for PrEP‐related testing when accessing PrEP via alternative PrEP providers and on‐demand PrEP use.

**Conclusions:**

Expanding PrEP coverage is crucial to reduce HIV acquisitions further and reach zero new acquisitions by 2030. As the Dutch National PrEP Programme reached capacity limits, PrEP expansion through alternative routes should be encouraged. Nevertheless, balancing out‐of‐pocket expenses and reimbursed care is key for healthcare equity.

## 1 INTRODUCTION

Ending the HIV epidemic by 2030 requires HIV prevention services to better reach and cover populations at risk. A highly effective prevention strategy is HIV oral pre‐exposure prophylaxis (PrEP), formally available since 2019 in the Netherlands. PrEP has emerged as a cornerstone in the Dutch HIV prevention arsenal, targeting primarily men who have sex with men (MSM) who align with the eligibility criteria [1, 2]. In 2022, a significant decline of 67% in new HIV acquisitions was seen among MSM compared to 2010 [3]. This notable reduction underscores the possibility of reaching the last mile of ending the HIV Epidemic [4], thereby solidifying the essential role of PrEP alongside other prevention strategies (e.g. treatment as prevention) in the HIV prevention strategies of the Netherlands.

Presently, most PrEP is administered via the Dutch National PrEP Programme within the sexual health centres affiliated with public health services [5]. However, in 2022, the National PrEP Programme had reached its maximum provision capacity (8500 slots). As a result, an estimated 3000 MSM cannot access PrEP services and are on the waiting list [6]. This limited National PrEP Programme's PrEP provision capacity, mostly due to the limited budget for PrEP provision, creates obstacles in PrEP uptake, compelling MSM to seek alternative routes via other alternative governmental and non‐governmental providers (hereinafter alternative PrEP providers), including but not limited to the general practitioners (GPs, governmental) or online clinics (non‐governmental), to bypass the lengthy waiting list. Consequently, roughly 4500 MSM are utilizing PrEP via alternative PrEP providers as estimated by the Dutch National Institute for Public Health and Environment. Both numbers are likely underrepresentations, and the current actual size of PrEP‐eligible/intending MSM lacking PrEP access is unknown.

Access via the National PrEP Programme offers several advantages. First, PrEP is offered with a subsidized co‐payment of €7.50 per month while other alternative PrEP providers request an out‐of‐pocket payment of >€30. Second, a comprehensive care package, without additional payment, is provided, including HIV, hepatitis C and other sexually transmitted infections (STIs) screening and kidney function testing. Lastly, a consistent PrEP supply is assured [5, 7]. On the contrary, PrEP access through alternative PrEP providers can result in out‐of‐pocket costs of up to approximately €300 every 3 months (including PrEP and PrEP‐related testing), contingent on prescribers and healthcare insurance reimbursements. The scarcity of available National PrEP Programme's PrEP slots, therefore, contributes to disparities in PrEP access, perpetuating health inequity among individuals with limited financial resources or other marginalized populations [7, 8, 9, 10]. Moreover, the National PrEP programme's co‐payment for PrEP will increase to €30 in 2024, further increasing this gap.

Although the Dutch Minister of Health decided to expand the PrEP care budget by €1 million per year (a 14% increase from 2022), given the high inflation rates in the Netherlands, professionals are concerned that this will only marginally extend the capacity of the National PrEP Programme [11, 12]. Consequently, a significant increase in the capacity of PrEP provision by the National PrEP Programme is unlikely, maintaining the existing challenge of PrEP access offered by the National PrEP Programme. A broader PrEP coverage and provision may only be achieved with a more substantial role of alternative PrEP providers.

Therefore, in this study, we first estimate the size of PrEP‐eligible MSM in the Netherlands. We then assess the epidemiological and health‐economic impact of expanding PrEP coverage by the National PrEP Programme and alternative PrEP providers, focussing on multi‐healthcare PrEP access to a commitment to ending the HIV epidemic and striving for health equity.

## 2 METHODS

### 2.1 Study population of PrEP‐eligible/intending MSM

We estimated the number of PrEP‐eligible/intending MSM living in the Netherlands, using a Dutch subsample of the European‐MSM‐Internet‐Survey (EMIS‐2017) [13, 14]. EMIS‐2017 recruited 3851 MSM living in the Netherlands between October 2017 and January 2018, of which 3232 were HIV negative [13]. Following the Dutch PrEP eligibility guidelines, HIV‐negative MSM who have had (1) condomless anal intercourses with partner(s) with unknown or seropositive HIV status, (2) syphilis, chlamydia or gonorrhoea, and/or (3) a post‐exposure prophylaxis prescription in the preceding 6 months are considered to be PrEP‐eligible [5]. EMIS‐2017 also requested information on the intention for PrEP use, defined as quite‐ and very‐likely to use PrEP when PrEP is available and affordable [14, 15].

### 2.2 Mathematical model assumptions and calibration

To project the epidemiological and health‐economic impact among MSM of 15 years and older, we used an existing and published transmission model of HIV among MSM in the Netherlands (schematic representation of the model, the equations and assortative sexual risk mixing matrix were published [16], and can be found in Supplementary S1), assuming a closed population. In short, our model stratifies disease progression into the acute stage, three chronic stages (CD4>500 cells/ml, CD4 count 350–500 cells/ml and CD4 count 200–349 cells/ml) and AIDS (CD4<200 cells/ml). Our model included four different sexual risk groups with different levels of sexual activity based on the annual number of new sexual partners [16, 17]. Epidemiological parameters included: the estimated size of the MSM population, the number of oral PrEP users, the number of MSM diagnosed with HIV, the estimated number of MSM living with HIV, the yearly number of new HIV diagnoses including the proportion diagnosed in a late/advanced stage and the proportion of HIV diagnosed MSM receiving antiretroviral treatment. The estimated HIV incidence in each sexual risk group were previously published [17], and are included in our Table S1.

The model was calibrated on epidemiological HIV data among MSM in the Netherlands from 2017 to 2021 (for key parameters and model calibration details, see Tables S1 and S2), and 117 of 500,000 simulations were accepted that best matched the epidemic [3]. The model starts on the first of January 2022, with a PrEP expansion scale‐up period until January 2024, assuming an effectiveness of 86% [18]. In the base‐case scenario (no PrEP expansion), the current Dutch PrEP provision is simulated (13,000 PrEP users of which 8500 are in the National PrEP Programme and 4500 via alternative PrEP providers by 2022). Several other scenarios were compared to the base‐case, including additional coverage of 3000 waiting‐list MSM, and in addition one‐third (5000), two‐thirds (10,000) and all (15,000) of PrEP‐eligible/intending MSM after 2 years, targeting MSM who belong to the medium‐high/high‐risk group.

### 2.3 Statistical analysis

#### 2.3.1 Epidemiological impact of PrEP expansion, regardless of the provider

We modelled the epidemiological impact based on new HIV acquisitions and cumulative HIV acquisitions averted from 2022 to 2030. In this epidemiological impact analysis, our model did not consider the different PrEP providers, given no data on PrEP use patterns, including effectiveness, adherence and discontinuation, between users accessing through the National PrEP Programme and alternative PrEP providers, was available in the Netherlands. Therefore, we assumed similar PrEP use patterns between users access through both pathways. Additionally, we explored the potential impacts of varying adherence levels, ranging from 69% effectiveness with suboptimal adherence to 93% effectiveness with high adherence [18]. We also assessed scenarios involving different PrEP delivery strategies, including targeted expansion to medium‐low/medium‐high/high‐risk groups, and universal access without differentiated targeting. All results are reported as the median of the accepted simulations, with interquartile ranges (IQRs).

#### 2.3.2 Cost‐effectiveness and budget impact analysis

In our model, each compartment was assigned a Dutch‐specific quality‐adjusted life year (QALY) score [19] and a cost. Healthcare resource unit prices were based on recommended unit prices by the Dutch Healthcare Institute and the Dutch Healthcare Authority [20]. The healthcare utilizations were based on clinical data from the Dutch MSM population, stratified by the diagnosed stages [21] (Tables S3 through S5 for detailed utility weighting and costs). Costs and QALY were discounted at 3% per year. Incremental cost‐effectiveness ratios (ICERs) were determined, using a Dutch willingness‐to‐pay threshold set at <€20,000 per QALY. The cost‐effectiveness was calculated using a payer's perspective, including only those monetary costs (e.g. treatment costs and other health service resource use associated with disease management) incurred by the Dutch National Health Insurance [22], with a lifetime horizon of 40 years from 2022 onwards, which was the estimated lifespan between the average age at HIV diagnosis and the male life expectancy in the Netherlands. In the cost‐effectiveness analysis, given the unknown and unsure future of PrEP expansion via the National PrEP Programme in the Netherlands, we assumed a stable National PrEP Programme PrEP provision capacity at 8500 slots, with the remaining PrEP prescribed and provided by alternative PrEP providers. All oral PrEP in this analysis was generic tenofovir disoproxil fumarate/emtricitabine.

Lastly, to explore the additional short‐term costs associated with PrEP expansion, a budget impact analysis and a net benefit analysis were performed over 5 years. In the budget impact analysis, we explored the total short‐term costs of the base‐case scenario and the additional net costs to both the payer and PrEP users associated with expanding PrEP provision only in the National PrEP Programme, ranging the National PrEP Programme's capacity from 8500 to 28,000 slots, covering all PrEP‐eligible/intending MSM in the Netherlands. In the net benefit analysis, we further accounted for the cumulative averting costs for new HIV acquisitions.

#### 2.3.3 One‐way sensitivity analysis

A one‐way sensitivity analysis was conducted to evaluate the cost‐effectiveness of expanding PrEP provision to all eligible and intending MSM, using the base‐case scenario as the comparator. Key input variables were varied, including QALY discounting (0–5%), cost discounting (0–10%), annual antiretroviral therapy (ART) costs (adjusted by ±30%) and the co‐payment for PrEP‐related testing by non‐governmental providers (ranging from €385 to €0, with €0 representing full coverage of testing costs). We also examined factors related to PrEP provision and its effectiveness. This included scenarios of higher/lower real‐world PrEP effectiveness due to users’ adherence and the use of on‐demand PrEP, which is common among MSM in the Netherlands [23]. Although the epidemiological impact of on‐demand versus daily PrEP is unlikely to differ significantly due to their similar efficacy [1, 24], lower pill utility was reported among on‐demand PrEP users [25], resulting in reduced PrEP‐related costs. To account for this, we modelled the economic implications if all PrEP users transitioned to on‐demand use, assuming pill utilization would decrease by 50% compared to daily PrEP use [25].

## 3 RESULTS

### 3.1 Estimated size of PrEP‐eligible/intending MSM

Based on the 3232 HIV‐negative MSM of the Dutch EMIS‐2017 subsample, we estimated that approximately 35% were PrEP‐eligible. Among those, approximately 45% indicated “quite‐” and “very‐likely” to use PrEP when PrEP is available and affordable. Assuming our estimates are generalizable to the Dutch general MSM population, based on the estimated HIV‐negative MSM size in the Netherlands [26], this results in approximately 28,000 PrEP‐eligible/intending MSM who would also benefit from PrEP, and approximately 15,000 PrEP‐eligible/intending MSM with unmet PrEP needs.

### 3.2 Epidemiological impact of PrEP expansion, regardless of the provider

**Figure 1:**
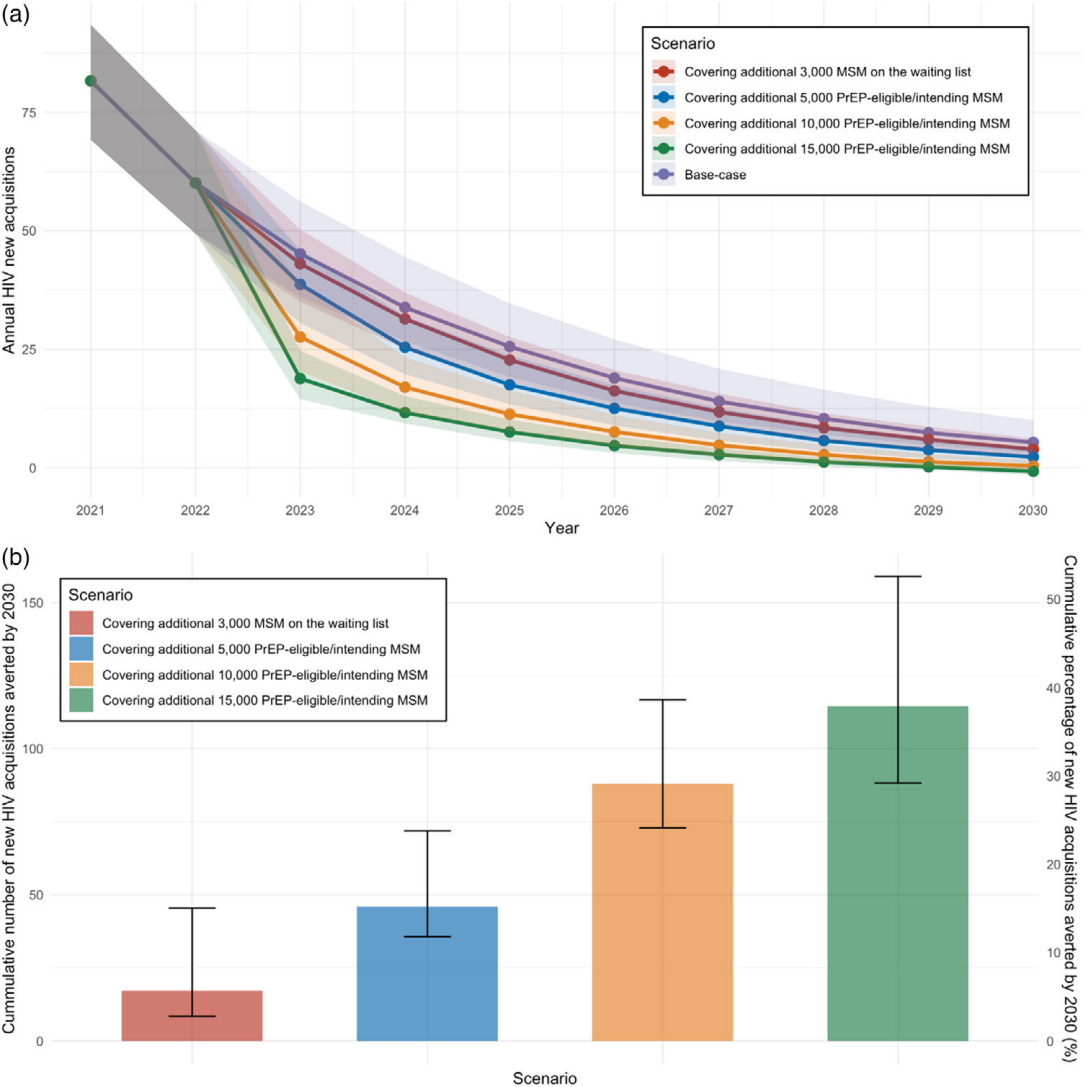
Projected (a) HIV new acquisitions, and (b) cumulative HIV new acquisitions averted by 2030 by further expanding PrEP provision to MSM who are currently on the waiting list, and MSM who are PrEP‐eligible and intend to use PrEP, the Netherlands, 2021–2030. *Note*: Lines show median across 117 fits. Shades show the interquartile range (IQR) of the median. Bars and error bars show the median and IQR across 117 fits.

In the based‐case scenario, an estimated cumulative 302 (IQR = 239−388) HIV acquisitions will occur between 2024 and 2030, with five (IQR = 3−10) new HIV acquisitions in 2030. Extending PrEP coverage to 3000 MSM on the waiting list, one‐third (5000), two‐thirds (10,000) and all (15,000) PrEP‐eligible/intending MSM by January 2024 will lead to a reduction in new HIV acquisitions, resulting in a total of 4 (IQR = 2−6), 2 (IQR = 1−4), 0 (IQR = 0−2) and 0 (IQR = 0−0) new acquisitions in 2030, respectively (Figure 1a). This translates to a cumulative reduction of 17 (IQR = 8−46, 5.66% [IQR = 2.18−19.04%]), 46 (IQR = 36−72, 15.18% [IQR = 9.20−30.06%]), 88 (IQR = 73−117, 29.08% [IQR = 18.80−48.87%]) and 115 (IQR = 88−159, 37.88 [IQR = 33.37−42.78%]) new acquisitions compared to the base‐case (Figure 1b). Although both covering two‐thirds and all PrEP‐eligible/intending MSM by January 2024 would both result in zero new HIV acquisition, covering all MSM has a higher cumulative reduction of new acquisitions and would reach zero new acquisitions sooner.

**Figure 2:**
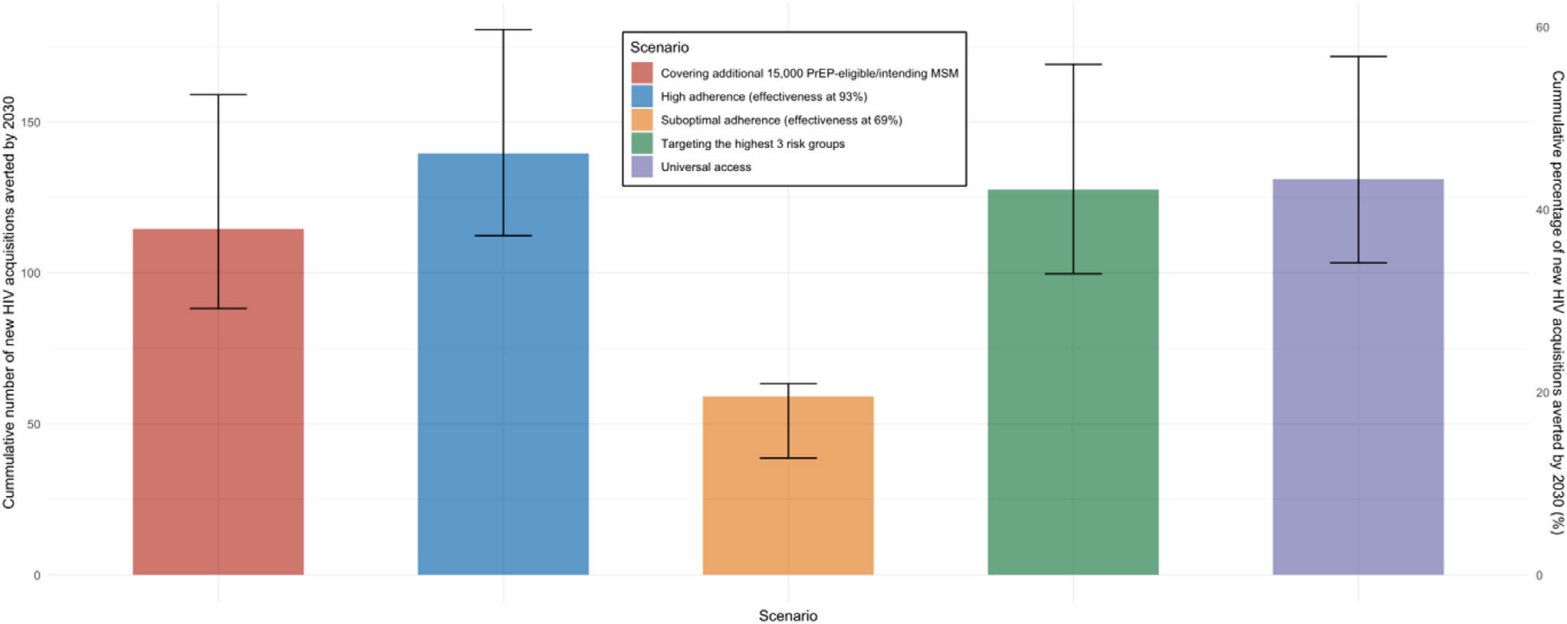
Projected cumulative HIV new acquisitions (percentage) averted by 2030 by further expanding PrEP provision to all MSM who are PrEP‐eligible and intend to use PrEP by different real‐world effectiveness due to adherence and different PrEP targeting strategies, the Netherlands, 2021–2030. *Note*: Lines show median across 117 fits. Bars and error bars show the median and interquartile range (IQR) across 117 fits.

The epidemiological impact of PrEP expansion is highly sensitive to the adherence of the PrEP users. Under the scenario of covering all PrEP‐eligible/intending MSM, high adherence results in a larger cumulative reduction in new acquisitions (140, IQR = 112−181, 46.16% [IQR = 28.98−75.58%]). In contrast, suboptimal adherence reduces this impact significantly, with a cumulative reduction of 59 (IQR = 39−63, 19.56% [IQR = 9.97−25.53%]) acquisitions by 2030. While the epidemiological impact is less sensitive to PrEP targeting strategies, the results favour universal access. When targeting medium‐low/medium‐high/high‐risk groups, the cumulative reduction would increase to 128 (IQR = 100−169, 42.20% [IQR = 25.72−70.76%]), when having universal access, the cumulative reduction would further increase to 131 (IQR = 103−171, 43.35% [IQR = 26.66−71.83%]) (Figure 2).

### 3.3 Cost‐effectiveness and budget impact analysis of PrEP expansion

In the base‐case scenario, the model estimates that the epidemic costs €6179 million and 6,343,507 QALYs until 2062. Covering all PrEP‐eligible/intending MSM costs €6172 million (−€7 million compared to the baseline) and gained QALYs (6,346,781, +3274 QALY), resulting in a cost‐saving scenario (−€2160/QALY). Although the other scenarios were dominated, PrEP provision expansion through alternative PrEP providers results in a reduction in cost and an increase in QALYs (Table 1).

**Table 1:** Cost‐effectiveness of expanding PrEP provision via alternative PrEP providers over the different scenarios, the Netherlands

PrEP provision scenarios/slots	Total costs, Euro's € (millions)	QALY (x1000)	Incremental costs compared to previous scenario Euro's € (millions)	Incremental QALYs compared to previous scenario	ICER	Cumulative HIV acquisitions averted by 2030 compared to the Base‐case scenario
13,000 (8500 via National PrEP Programme + 4500 via alternative PrEP providers) (Base‐case)	€ 6178.92	6343.51	−	−	−	−
28,000 (Expansion of 15,000)	€ 6171.84	6346.78	**€** −**7,08**	**3274**	Cost‐saving	115
23,000 (Expansion of 10,000)	€ 6172.36	6346.74	**€ 0,52**	−**36**	Dominated^a^	88
18,000 (Expansion of 5000)	€ 6173.92	6346.03	**€ 1,56**	−**718**	Dominated^a^	46
16,000 (Expansion of 3000)	€ 6175.98	6345.25	**€ 2,06**	−**779**	Dominated^a^	17

Note: The reported numbers are median across 117 fits. PrEP expansion is through MSM on the waiting list or PrEP eligible/intending MSM. For the cost‐effectiveness scatterplot, see Figure S2.

Abbreviations: ICER, incremental cost‐effectiveness ratio; MSM, men who have sex with men; PrEP, pre‐exposure prophylaxis; QALY, quality adjusted life years.

^a^ Dominated indicates when the compared strategy has equal or less QALYs compared with the previous less costly scenario.

Without any PrEP provision expansion, our budget impact analysis projected a total overall cost of €23 million for the payers (the government and the National PrEP Programme) and €15 million in out‐of‐pocket expenses for PrEP users. For the payers, our model projected additional costs from €6.0 to €45.3 million, while the net benefits of €1.9−€45.0 million were projected over 5 years if the National PrEP Programme's PrEP provision capacity increases from 10,000 to 28,000 slots, respectively. Conversely, as the total number of PrEP accessed via the alternative PrEP providers decreases, the additional costs to the PrEP users decrease from €38.8 million to −€6.5 million over 5 years, respectively (Table 2).

**Table 2:** Budget impact and net benefits of increasing National PrEP Programme's PrEP provision capacity to additional cover all (15,000) PrEP eligible/intending MSM

		Provision provider contribution (slots)		5‐year additional costs of PrEP expansion compared to the Base‐case scenario (millions)^a^		Cumulative HIV acquisitions averted compared to the Base‐case scenario		
National PrEP Programme's capacity scenarios	Total PrEP coverage (slots)	National PrEP Programme	Alternative PrEP providers	National payer (Government)	PrEP users	5 years	by 2030	5‐year net benefit (millions)^b^
Base‐case	13,000	8500	4500	0	0	0	0	−
8500 slots	28,000	8500	19,500	€ 2,25	€ 38,75	92	115	−**€ 1,94**
10,000 slots	28,000	10,000	18,000	€ 5,97	€ 35,02			−**€ 5,66**
15,000 slots	28,000	15,000	13,000	€ 18,33	€ 22,61			−**€ 18,02**
20,000 slots	28,000	20,000	80,00	€ 30,63	€ 10,19			−**€ 30,32**
25,000 slots	28,000	25,000	30,00	€ 42,76	−**€ 2,23** ^c^			−**€ 42,45**
28,000 slots	28,000	28,000	0	€ 45,29	−**€ 6,54** ^c^			−**€ 44,98**

Note: The reported numbers are median across 117 fits.

Abbreviation: PrEP, pre‐exposure prophylaxis.

^a^ Budget impact over 5 years of the additional costs for the PrEP care continuum, calculated as the total costs of the PrEP care continuum per person*the total number of PrEP users.

^b^ Net benefit was calculated as the costs of the additional PrEP care continuum over 5 years—the cumulative costs of averted HIV acquisitions over 5 years.

^c^ Costs were saved due to the total number of PrEP accessed via the alternative PrEP providers decreases (less individual co‐payments).

### 3.4 One‐way sensitivity analysis

**Figure 3:**
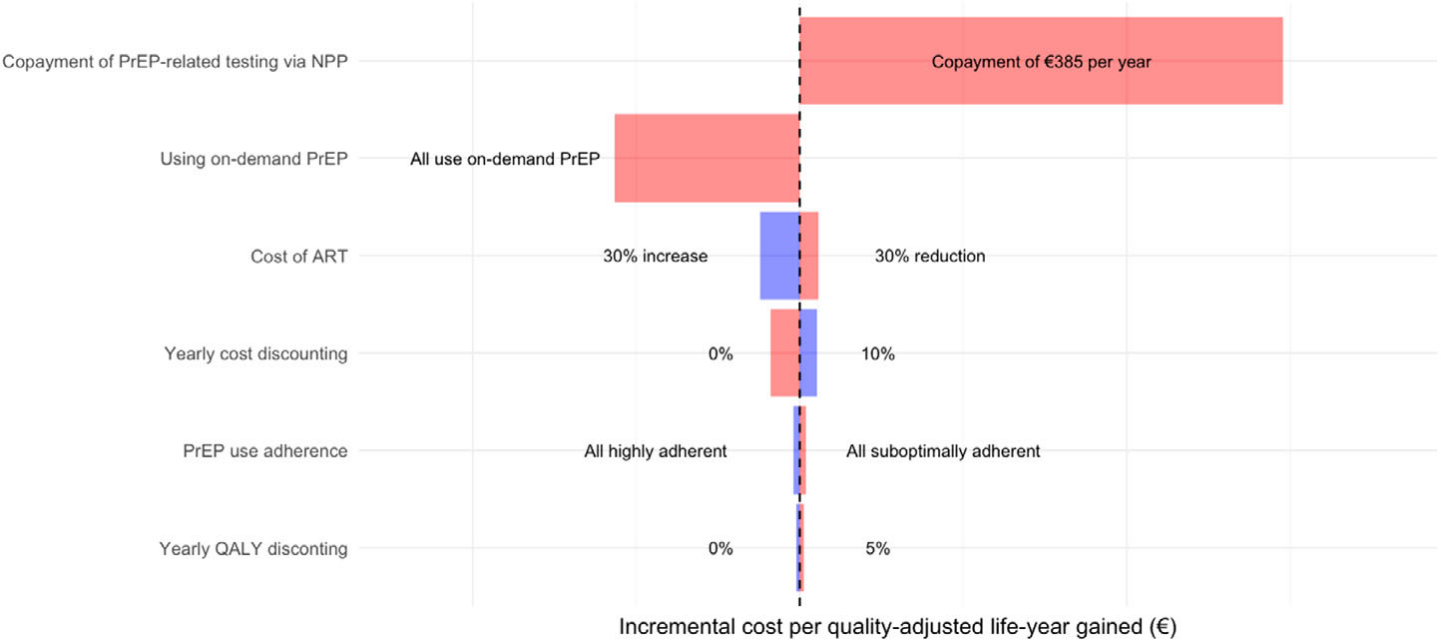
One‐way sensitivity analyses of the incremental cost‐effectiveness of improving PrEP provision to cover all of PrEP‐eligible/intending MSM compared with the current PrEP provision in the Netherlands over 40 years, 2022–2062. *Note*: Lines show median across 117 fits. Abbreviations: ART, antiretroviral treatment; NPP, National PrEP Programme; PrEP, pre‐exposure prophylaxis; QALY, quality adjusted life years.

In the one‐way sensitivity analysis, we found that our cost‐effectiveness analysis is most sensitive to the individual co‐payment for PrEP‐related testing when accessing PrEP via alternative PrEP providers. Reducing the yearly individual co‐payment from €385 to €0 will significantly increase the costs and lead to an ICER of €72,000 per QALY. Following the individual co‐payments, on the opposite, our cost‐effectiveness analysis is second‐most sensitive to on‐demand PrEP use. Assuming all PrEP users would use on‐demand PrEP instead will significantly decrease the costs and lead to an ICER of −€30,520. In addition, our cost‐effectiveness analysis is also sensitive to the yearly cost discounting, and yearly cost of ART and real‐world PrEP effectiveness due to adherence, while other key input variables showed limited influence on the projected cost‐effectiveness (Figure 3).

## 4 DISCUSSION

This study reports both the epidemiological and health‐economic impact of expanding PrEP provision in the Netherlands. Our model forecasts the potential to achieve zero new HIV acquisitions among Dutch MSM by 2030 through the expansion of the current PrEP programme and increasing the role of alternative PrEP providers. However, it is important to note that achieving zero new HIV acquisitions does not equate to zero new HIV diagnoses, as late or advanced diagnoses remain prevalent in the Netherlands. Sustained HIV prevention efforts are essential to reach marginalized and underserved populations and regions [27, 28, 29, 30]. Further, our sensitivity analysis highlights the critical role of PrEP adherence in maximizing effectiveness and supports universal access beyond risk‐based targeting to amplify the epidemiological impact of oral PrEP expansion. More importantly, our findings showed that PrEP expansion can be cost‐saving if all PrEP‐eligible/intending MSM were covered additional via alternative PrEP providers. This study thus underscores the advantages of increasing PrEP provision in substantially reducing HIV acquisitions among MSM and reaching zero by 2030, aligning with the substantial epidemiology impact of PrEP on the HIV epidemic described in previous modelling studies in the Dutch context [17, 31, 32]. Our results are, therefore, not only timely and valuable for the Dutch discourse on PrEP expansion but also carry relevance for other European countries with similar HIV epidemics and PrEP service provision structures.

Yet, obtaining the ideal epidemiological and health‐economic impact, as projected by our model, can be challenging given the current paradox of the Dutch PrEP access and provision due to the lengthy wait list of the National PrEP Programme and the financial burdens to PrEP user when accessing via alternative PrEP providers. Additionally, beyond the financial burdens to PrEP users, alternative PrEP providers, for example GPs, might also be reluctant to PrEP prescription. Reasons include perceiving PrEP provision as beyond their usual purview [7], lower knowledge of HIV and PrEP [7], or HIV‐related or PrEP‐related stigma [33]. This would likely lead to a lower‐than‐expected willingness for PrEP prescription and, hence, less provision. Yet, given that alternative PrEP providers, especially GPs, usually have closer connections with their patients compared to the sexual health centres where the National PrEP Programme is embedded, encouraging a more substantial role for GPs in PrEP provision is thus essential, and could help reach MSM facing barriers to accessing PrEP services, especially those lacking information and accessibility to PrEP services [7, 34]. Decentralization of PrEP provision may only succeed by providing more training and education on PrEP and its relevance in HIV prevention, and by offering more (financial) incentives to alternative PrEP providers. A similar approach has increased PrEP uptake in France, a similar setting as the Netherlands [15, 35].

Additionally, in our cost‐effectiveness analysis, the assumption that alternative PrEP providers contribute additional PrEP beyond the National PrEP Programme's capacity means that the costs associated with PrEP expansion in our model largely fall on PrEP users rather than being state‐funded. This results in higher individual co‐payments and minimal costs to the payers. With a lower cost associated with treatments for the new HIV acquisition due to the higher cumulative averted HIV new acquisition, such a cost‐saving estimate was expected. However, the current PrEP provision over alternative PrEP providers with larger out‐of‐pocket expenses would also make PrEP difficult to access for MSM, especially among those who are not accessing the National PrEP Programme, which are often part of marginalized populations, including migrants and MSM with ethnic minority self‐identification or lower socio‐economic positions [8, 13]. Therefore, allocating a higher budget towards the National PrEP Programme as estimated and outlined in our budget analysis and net benefit analysis, or making the current National PrEP Programme more cost‐efficient to enlarge its PrEP provision capacity seems indicated and necessary. Consequently, more MSM, particularly individuals from marginalized groups, would have more equitable access.

To do so, PrEP‐prioritizing criteria additionally to the current PrEP eligibility criteria guideline can be warranted. For example, to prioritize MSM who are financially struggling, their financial subsidy uptake status (e.g. Bijstandsuitkering, a subsidy that is only available for those who are financially extremely disadvantaged) can be referred to evident the PrEP prioritization process. This approach aligns with the call for the End Inequalities initiative by UNAIDS [36], and thus deserves endorsement. Another approach could be better‐integrated PrEP‐related screening at the sexual health centres. Serving as the primary routine STI testing provider in the Netherlands, the sexual health centres delivered 72,210 STI consultations to MSM in 2022, with only 39% (27,892 consultations) directed towards MSM using PrEP through the National PrEP Programme [37]. However, PrEP‐related testing and the routine STI screening for MSM often overlap. If a better integrated PrEP and STI consultations can be embedded during the STI consultations among MSM, especially among MSM who are not included in the National PrEP Programme but showed intention to use PrEP, there would be potential for an expanded National PrEP Programme's PrEP provision capacity within the current National PrEP Programme budget. Also, our one‐way sensitivity analysis showed that one of the key parameters influencing cost‐effectiveness is the cost of STI testing. Therefore, to reduce the cost related to PrEP use, different PrEP‐related testing strategies should be considered. For instance, HIV self‐tests for those with limited or lower risk [38], or less frequent PrEP‐related testing (once every 6 months) [32, 39]. Lastly, another approach could centre on a centralized PrEP medication supply. Dutch policymakers should consider establishing a national centralized PrEP registry and medication distribution system. This would ensure a stable and affordable PrEP medication supply, with similar co‐payments for all PrEP users. In turn, MSM receiving PrEP prescriptions from both the National PrEP Programme and alternative PrEP providers can equally benefit from a stable national supply of PrEP provision, alleviating concerns about PrEP availability and different costs of PrEP medication.

Our work has several limitations. First, we used a similar effectiveness for MSM with PrEP administrated via the National PrEP programme and alternative PrEP providers, neglecting potential differences in adherence or discontinuation, as precise data on the current PrEP usage patterns between the users accessing via different PrEP providers is lacking. Second, our model assumed a closed population among MSM, overlooking the sudden new HIV acquisitions due to unusual migration movements such as one observed by the war refugees from Ukraine [40], as well as other high‐risk populations underserved by PrEP, such as trans* individuals [29]. Consequently, our model's projections were rather optimistic and could be sensitive to uncertainties that were not considered in our model, and may not be generalized to a broader population. Lastly, our model did not include the potential PrEP provision expansion via the National PrEP Programme, as planned in the summer of 2024, in our cost‐effectiveness analysis given the new PrEP provision capacity remains unknown. However, as this expansion would only be marginally extended with the additional budget [11, 12], our assumption of the continued significant contribution of alternative PrEP providers remains valid. Consequently, our cost‐effectiveness analysis may be an overestimation but would not be significantly biased. Furthermore, our budget impact analysis has outlined the short‐term cost for different PrEP provision capacities of the National PrEP Programme, thereby bolstering informed decision‐making with the upcoming PrEP provision expansion in the Netherlands. Finally, the absence of comprehensive sensitivity analyses on key parameters (particularly treatment cascade assumptions) represents an important limitation. Such analyses would have strengthened our understanding of how variations in these key parameters impact on the robustness of our findings, and, therefore, should not be overlooked in future analyses.

## 5 CONCLUSIONS

In conclusion, we advocate that policymakers should further expand the current PrEP provision in the Netherlands and other countries with similar HIV epidemics but also with limited PrEP provision capacities. The goal should be to cover as many PrEP‐eligible/intending MSM as possible, considering the projected potential to achieve zero HIV acquisitions among MSM by 2030. Moreover, we emphasize the need for Dutch policymakers to decentralize PrEP provision by actively encouraging alternative PrEP providers to play a more significant role and ensure a consistent PrEP medication supply to MSM, as this approach promises enhanced cost‐effectiveness from a payer's perspective. However, it is equally crucial to recognize that increasing the National PrEP Programme's capacity is indispensable for ensuring affordable and stable access for PrEP users. Therefore, we recommend achieving a delicate balance between the costs to the payer and the individual. Further, we also want to stress the importance of extending PrEP services and other HIV prevention coverage among other key populations affected by HIV, such as heterosexual women (especially with a migrant background), trans* individuals and sex workers to ensure reaching zero new HIV acquisitions. However, we must also acknowledge the challenges posed by declining global funding for HIV prevention and the growing resistance to diversity, equity, and inclusion initiatives worldwide. In this context, it is crucial to safeguard existing resources and budgets for HIV prevention to ensure preparedness for unforeseen challenges that could hinder progress towards ending the HIV epidemic. In summary, we urge policymakers to prioritize making PrEP accessible and available to all PrEP‐eligible and PrEP‐intending MSM in the Netherlands, regardless of the provider. The time is now.

## COMPETING INTERESTS

HW, DvdV and KJJ report grants from ViiV Healthcare outside the submitted work. DvdV and KJJ also report grants from Gilead Sciences outside the submitted work. SP reports no conflict of interest

## AUTHORS’ CONTRIBUTIONS

HW and KJJ conceptualized this research. HW, SP, DvdV and KJJ collected the data for this research. HW and SP analysed the data. HW drafted the manuscript. All authors critically revised the manuscript for intellectual content. All authors read and approved the final version of the manuscript.

## FUNDING

There was no funding source for this study.

## Supporting information




**Figure S1**: Schematic representation of the compartmental deterministic model. The state variables used in the equations are shown between brackets.
**Figure S2**: Cost‐effectiveness scatter plot.
**Table S1**: Key model parameters of PrEP for HIV prevention in the Netherlands.
**Table S2**: Variables used to calibrate and accept simulation using Latin Hypercube sampling techniques.
**Table S3**: Assumed utility weighting for Quality‐adjusted life year (QALY).
**Table S4**: Annual costs per service for the use of oral pre‐exposure prophylaxis (PrEP) in the Netherlands from a payer's perspective.
**Table S5**: Annual costs per service for the antiretroviral drugs and other direct healthcare costs by diagnosed stage in the Netherlands from a payer's perspective.

## Data Availability

The data that has been used for calibration and validation of the model is available in Tables S1 and S2 in the supplement. The model codes are publicly available at OSF: DOI 10.17605/OSF.IO/8UXPN.
