# Supplementary material for: Epidemiological impact and cost‐effectiveness analysis of PrEP provision expansion among MSM in the Netherlands
Source: J Int AIDS Soc. 2025 Jun 3;28(6):e26516. doi: 10.1002/jia2.26516 (PMC12134395; doi:10.1002/jia2.26516)
Supplement: Supplementary file 1 — Figure S1: Schematic representation of the compartmental deterministic model. The state variables used in the equations are shown between brackets. Figure S2: Cost‐effectiveness scatter plot. Table S1: Key model parameters of PrEP for HIV prevention in the Netherlands. Table S2: Variables used to calibrate and accept simulation using Latin Hypercube sampling techniques. Table S3: Assumed utility weighting for Quality‐adjusted life year (QALY). Table S4: Annual costs per service for the use of oral pre‐exposure prophylaxis (PrEP) in the Netherlands from a payer's perspective. Table S5: Annual costs per service for the antiretroviral drugs and other direct healthcare costs by diagnosed stage in the Netherlands from a payer's perspective. [file JIA2-28-e26516-s001.docx]

**Epidemiological impact and cost-effectiveness analysis of expanding PrEP provision to MSM who are PrEP-eligible expressing PrEP-intention in the Netherlands:**

**Supplementary S1 File**

Haoyi Wang^1,2*^, Stephanie Popping^3,4,5^, David van de Vijver^2^, Kai. J. Jonas^1^

^1^Department of Work and Social Psychology, Maastricht University, Maastricht, The Netherlands

^2^Viroscience department, Erasmus Medical Centre, Rotterdam, The Netherlands

^3^Department of medical microbiology and infection prevention, Amsterdam University Medical Center, University of Amsterdam, The Netherlands

^4^Cener for infection and Molecular Medicine (CIMM), Amsterdam University Medical Center - location AMC, Amsterdam, the Netherlands

^5^Amsterdam Institute for Immunology and Infectious Diseases, Amsterdam, the Netherlands

* Correspondence to: Haoyi Wang

Email: haoyi.wang@maastrichtuniversity.nl

# S1 Mathematical transmission model

This study includes a compartmental deterministic mathematical transmission model that was developed for the HIV epidemic among men who have sex with men (MSM) in the Netherlands and Germany [1, 2]. The schematic representation of the model is presented in figure S1.

**Figure S1 – Schematic representation of the compartmental deterministic model. The state variables used in the equations are shown between brackets.**


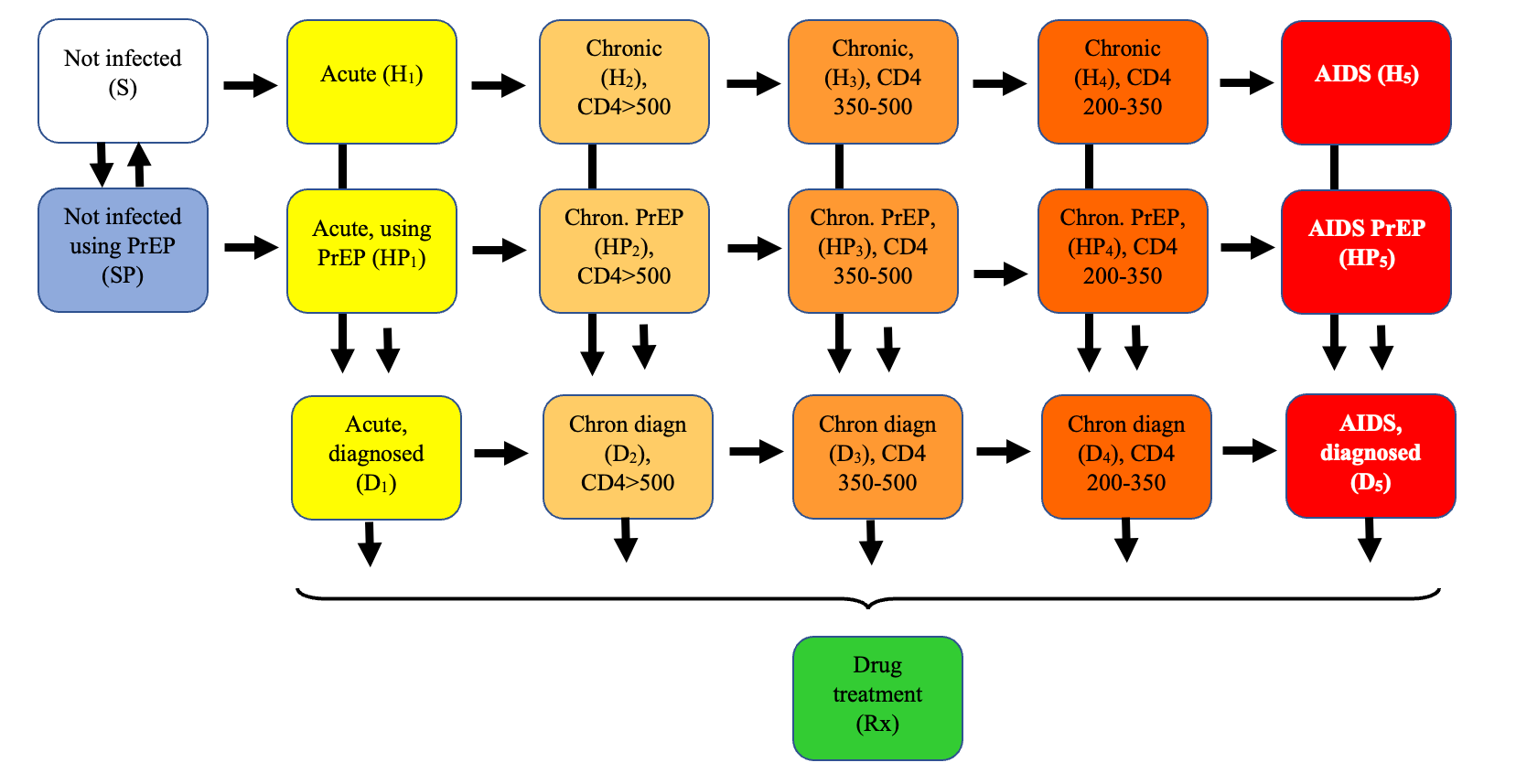


## Equations of the transmission model

The model consists of 15 ordinary differential equations, including two equations that describe individuals that are not infected with HIV that use PrEP or do not use PrEP, ten equations that describe disease progression, two equations that describe the force of infection (or the rate by which individuals become infected)[3] and one equation that describes mixing between individuals of different risk groups[3, 4]. The equations are summarized below.

### Ordinary differential equations for people not infected with HIV

Individuals not infected with HIV are sub-divided into individuals that are not on PrEP, denoted as S (susceptible individuals modelled using equation 1) and individuals using PrEP denoted as SP (suspeptible individuals using PrEP, modelled using equation 2). The sexual activity classes are defined in the equations in four groups *a* ranging from the group with the highest sexual activity (*a* = 1) to the group with the lowest sexual activity (*a* = 4). Susceptible individuals have a mortality rate *μ.* Individuals that become sexually active enter the group of susceptible individuals not using PrEP at a rate *Γ* times the proportion *π_a_* of each sexual activity group *a.* Values for the parameter *Γ* have been calibrated to the German MSM population[5] (Table S1). Susceptible individuals can start PrEP at a rate *Θ_a_* and discontinue PrEP at a rate *Φ_a_* (the values *Θ_a_* and *Φ_a_* for the lowest sexual activity groups *a=3* and *a=4* were set to zero as we assumed that PrEP will only be used for MSM at high risk for HIV infection). The force of infection, denoted as *λ_a_* for individuals not using PrEP (modelled using equation 13) and *λ^P^_a_* for individuals using PrEP (equation 14), represents the rate by which individuals become infected with HIV.

S_a_’ = Γ.π_a_ – S_a_ (λ_a_ + Θ_a_ + µ) + SP_a_.Φ_a_ (1)

SP_a_’ = S_a_.Θ_a_ - SP_a_(λ^P^_a_ + Φ_a_ + µ) (2)

### Equations for undiagnosed HIV infected individuals

Individuals that become infected with HIV but that have not been diagnosed yet (denoted with *H* in equations 3 through 5), progress through five stages of infection that are denoted by the parameter *σ*. These stages of infection are the acute stage (*σ* = 1, modelled using equation 3), the chronic stages (*σ* = 2 if the CD4 cell count is > 500 cells/µl, *σ* = 3 for a CD4 between 350 and 500 cells/µl and *σ* = 4 for a CD4 between 200 and 350 cells/µl, all modelled using equation 4) and the AIDS stage (*σ* = 5, modelled using equation 5). Individuals progress through infection at a rate *γ_σ_* that depends on the stage of infection *σ*_[6]_. Individuals are diagnosed at a rate *δ_σ_* that has been calibrated to the proportion of MSM that are diagnosed at a particular CD4 threshold as reported by the Robert Koch Institute. The mortality rate depends on the stage of infection (denoted as *µ_σ_*).

H_σ,a_’ = λ_a_.S_a_ - H_σ,a_(δ_σ_ + γ_σ_ + µ_σ_)….. for σ = 1 (3)

H_σ,a_’ = H_σ-1,a_.γ_σ-1_ - H_σ,a_(δ_σ_ + γ_σ_ + µ_σ_)….. for σ = 2, 3, 4 (4)

H_σ,a_’ = H_σ-1,a_.γ_σ-1_ - H_σ,a_(δ_σ_ + µ_σ_)………..for σ = 5 (5)

### Equations for HIV infected undiagnosed individuals using PrEP

The equations used to model individuals that become infected with HIV despite the use of PrEP (denoted as *HP* in equations 6 through 8), are comparable to the equations used to model HIV-infected individuals that do not use PrEP (equations 3, 4 and 5). Individuals using PrEP are assumed to be tested for HIV every six months at a rate (denoted as *δ^P^*).

HP_σ,a_’ = λ^P^_a_.SP_a_ - HP_σ,a_(δ^P^ + γ_σ_ + µ_σ_)….. for σ = 1 (6)

HP_σ,a_’ = HP_σ-1,a_.γ_σ-1_ - HP_σ,a_(δ^P^ + γ_σ_ + µ_σ_)….. for σ = 2, 3, 4 (7)

HP_σ,a_’ = HP_σ-1,a_.γ_σ-1_ - HP_σ,a_(δ^P^ + µ_σ_)………..for σ = 5 (8)

### Equations for HIV untreated infected individuals that have been diagnosed

Individuals that are diagnosed with HIV but that are not treated with antiretroviral drugs (yet) are denoted as *D* (equations 9 through 11). Diagnosed individuals start treatment at a rate *ρ_σ_*. The rate *ρ_σ_* depends on the stage of infection *σ* to reflect past changes in the CD4 cell count at which treatment was initiated. Per recent treatment guidelines, antiretroviral drugs are started irrespective of the stage of infection as of 2017 when we assumed that PrEP became available in Germany.

D_σ,a_’ = H_σ,a_.δ_σ_. + HP_σ,a_.δ^P^ - D_σ,a_(γ_σ +_ ρ_σ_ + µ_σ_) ….. for σ = 1 (9)

D_σ,a_’ = H_σ,a_. δ_σ_.+ HP_σ,a_.δ^P^ + γ_σ-1_.D_σ-1,a_ - D_σ,a_(γ_σ +_ ρ_σ_ + µ_σ_) ….. for σ = 2, 3, 4 (10)

D_σ,a_’ = H_σ,a_. δ_σ_.+ HP_σ,a_.δ^P^ + γ_σ-1_.D_σ-1,a_ - D_σ,a_(ρ_σ_ + µ_σ_) ….. for σ = 5 (11)

### Equations for individuals using antiretroviral drug treatment

Individuals that use antiretroviral drugs are represented by the state variable *Rx* (equation 12). People treated with antiretroviral drug treatment are assumed to have the same mortality as the general population[7].

$Rx_{a}^{'}= \sum_{\sigma=1}^{5} \rho_{\sigma}D_{\sigma,a}-Rx_{a}\mu$ (12)

### Equations for the force of infection

The force of infection is modelled using equation 13 for individuals not using PrEP (*λ_a_*), and using equation 14 for individuals using PrEP (λ^P^_a_). The force of infection depends on the rate of sexual partner change (*c_a_*) for individuals with sexual activity *a*, and the probability by which these individuals form a sexual relationship with an individual with sexual activity *i* as determined by the mixing matrix *M_a,i_* (equation 15). The rate of infection also depends on the infectivity of the different stages of infection represented. The model uses three different parameters of infectivity that all depend on the stage of infection *σ*, including β_σ_ for untreated HIV infected individuals that do not use PrEP, β_σ_^P^ for individuals using PrEP and β_Rx_ for individuals using antiretroviral drug treatment.

λ_a_ = c_a_ $\sum_{i=1}^{4} \frac{Ma,i}{Ni}$ ($\sum_{\sigma=1}^{5} \beta$_σ_H_σ,i_ + $\sum_{\sigma=1}^{5} \beta$_σ_^P^HP_σ,i_ + $\sum_{\sigma=1}^{5} \beta$_σ_D_σ,i_ + β_Rx_Rx_i_) (13)

λ^P^_a_ = c_a_ $\sum_{i=1}^{4} \frac{Ma,i}{Ni}$ ($\sum_{\sigma=1}^{5} \beta$_σ_^P^H_σ,i_ + $\sum_{\sigma=1}^{5} \beta$_σ_^P^HP_σ,i_ + $\sum_{\sigma=1}^{5} \beta$_σ_^P^D_σ,i_ + β_Rx_^P^Rx_i_) (14)

### Mixing matrix

M_a,j_ is a mixing matrix in which the elements *a,j* are the probability that an individual in sexual activity class *a* forms a sexual partnership with an individuals with sexual activity *j*. The mixing matrix includes a factor *ε* which denotes the degree of assortative mixing, and δ_a,j_ denotes Kronecker delta which is equal to zero if individuals are in the same sexual activity class or equal to one if the individuals are in different sexual activity classes[4].

M_a,i_ = ε.δ_a,i_ + (1 – ε) c_a_  $\frac{N_{i}}{\sum_{a=1}^{4} c_{a}n_{a}}$ (15)

# S2 Model calibration

We calibrated our model to the historical epidemic based on: the estimated size of the MSM population, the number of oral PrEP users, the number of MSM diagnosed with HIV, the estimated number of MSM living with HIV, the yearly number of new HIV diagnoses including the proportion diagnosed in a late stage (CD4<350 cells/mm^3) and advanced stage of infection (CD4<200 cells/mm^3), and the proportion of HIV diagnosed MSM receiving antiretroviral treatment. Key model parameters can be found in Table S1 and variables that were used for model calibration can be found in Table S2. Latin Hypercube sampling was used to calibrate the model.

Table S1. Key model parameters of PrEP for HIV prevention in the Netherlands

| **Model parameters** | | **Estimate or range^a^/IQR** | **Reference** |
| --- | --- | --- | --- |
| Duration of disease stages | |  |  |
|  | Acute stage | 10–16 weeks | [8] |
|  | CD4+ T-cell count 350–500 cells/µL | 2.9–3.1 years | [9] |
|  | CD4+ T-cell count 200–349 cells/µL | 3.6–3.9 years | [9] |
|  | CD4+ T-cell count < 200 cells/µL | 13–25 months | [9] |
| Infectivity per partnership transmissibility per year | |  |  |
|  | Acute stage | 0.030–0·61 | [10]; Model calibration |
|  | Chronic stage | 0.027–0·21 | [10]; Model calibration |
|  | AIDS stage | 0.008–0·27 | [10]; Model calibration |
| Proportion MSM in sexual risk groups | |  |  |
|  | Highest | 11% (11-12% IQR) | Model calibration, the sum of the three groups was equal to 100% |
|  | 2^nd^ highest | 14% (12-16% IQR) |  |
|  | 3^rd^ highest | 19% (14-23% IQR) |  |
|  | Lowest | 56% (49-61% IQR) |  |
| Number partners/2 years in sexual risk groups | |  |  |
|  | Highest | 43.88 (34.95-55.27 IQR) | Model calibration |
|  | 2^nd^ highest | 9.78 (7.99-12.05 IQR) |  |
|  | 3^rd^ highest | 2.65 (1.85-3.67 IQR) |  |
|  | Lowest | 0.20 (0.14-0.29 IQR) |  |
| HIV incidence in sexual risk groups (incidence/100 person years) | |  |  |
|  | Highest | 0.46 | Model calibration |
|  | 2^nd^ highest | 0.10 |  |
|  | 3^rd^ highest | 0.03 |  |
|  | Lowest | 0.006 |  |
| Mortality rates per year | |  |  |
|  | Population | 0.0155 | [11] |
|  | Chronic HIV stage | 0.114 | [11] |
|  | AIDS stage | 0.648 | [11] |
|  | On treatment | 0.0184 | [11] |
| PrEP effectiveness | |  | [12] |
|  | Overall | 86% |  |
|  | High adherence | 93% |  |
|  | Suboptimal adherence | 69% |  |
| PrEP regimens discontinuation rate | | 0.62/year (0.48-0.79 IQR) | Model calibration |
| Primary cost parameters (costs listed are in 2022 euros) from a payer’s perspective | | | |
| Yearly cost of PrEP provided by the National PrEP Programme^b^ | | €698.19 | [13, 14]; Local data |
| Yearly cost of PrEP provided by alternative PrEP providers^b^ | | €43.19 | [13, 14]; Local data |
| Yearly cost of ART by diagnosed stages^c^ | |  |  |
|  | Timely presenter | €12465.92 | [13, 15] |
|  | Late presenter | €14541.90 | [13, 15] |
|  | Advanced presenter | €24372.57 | [13, 15] |

^Note: Abbreviations: AIDS: acquired immunodeficiency syndrome; HIV: human immunodeficiency virus; IQR: interquartile range; PrEP: pre-exposure prophylaxis.^

^a All ranges were uniformly distributed. b Details see Supplementary Table S3 c Details see Supplementary Table S4^

**Table S2 Variables used to calibrate and accept simulation using Latin Hypercube sampling techniques.**

| **Parameter used for calibration** | | | **Data in real world** | **Final values accepted in calibration range** | **Ranges in the accepted simulations (median, IQR)** | **Source** |
| --- | --- | --- | --- | --- | --- | --- |
| Dutch MSM population | | |  |  |  | [16] |
|  | 2017 | | 131515 | 120000 - 180000 | 161493 (156215 - 171141) |  |
|  | 2018 | | 133000 | 120000 - 180000 | 162002 (156622 – 171841) |  |
|  | 2019 | | 133515 | 120000 - 180000 | 162543 (157039 - 172553) |  |
|  | 2020 | | 135000 | 130000 - 200000 | 163076 (157465 - 173292) |  |
|  | 2021 | | 140000 | 130000 - 200000 | 163698 (157886 - 174982) |  |
| The number of oral PrEP users 2022 | | | 13000  (8500 via National PrEP Programe +  4500 via alternative providers) | 10000 - 15000 | 13015 (9336 - 16124) | [17, 18] |
| Number of MSM diagnosed with HIV | | |  |  |  | [19] |
|  | | 2017 | 12378 | 11500 - 14000 | 13439 (12911 - 13991) |  |
|  | | 2018 | 12697 | 11500 - 14500 | 13574 (13049 - 14219) |  |
|  | | 2019 | 12985 | 12000 - 14500 | 13658 (13129 - 14247) |  |
|  | | 2020 | 13332 | 12500 - 15000 | 13681 (13121 - 14227) |  |
|  | | 2021 | 13876 | 12500 - 15000 | 13652 (13112 - 14216) |  |
| The estimated number of MSM living with HIV | | |  |  |  | [19] |
|  | | 2017 | 14200 | 12000 - 16000 | 14778 (14287 - 15386) |  |
|  | | 2018 | 14200 | 12000 - 16000 | 14693 (14142 - 15303) |  |
|  | | 2019 | 14400 | 12000 - 16000 | 14560 (13966 - 15153) |  |
|  | | 2020 | 14500 | 12000 - 16000 | 14388 (13794 - 14991) |  |
|  | | 2021 | 14500 | 12000 - 16000 | 14139 (13566 - 14718) |  |
| Number of new diagnosis among MSM | | |  |  |  | [19] |
|  | | 2017 | 516 | 350 - 650 | 536 (491 - 575) |  |
|  | | 2018 | 437 | 300 - 600 | 511 (479 - 533) |  |
|  | | 2019 | 355 | 200 - 500 | 416 (392 - 434) |  |
|  | | 2020 | 340 | 200 - 500 | 340 (321 - 355) |  |
|  | | 2021 | 257 | 100 - 400 | 272 (255 - 284) |  |
| Proportion late diagnosis in 2021 | | | 42% | 37% - 47% | 42.8% (40.0% - 44.5%) | [19] |
| Proportion advanced diagnosis in 2021 | | | 22% | 17% - 27% | 20.1% (18,7% - 23.4%) | [19] |
| The proportion of HIV diagnosed MSM receiving ART | | |  |  |  | [19] |
|  | | 2017 | 91% | 85% - 95% | 93.9% (92.8% - 94.7%) |  |
|  | | 2018 | 93% | 87% - 98% | 95.1% (94.4% - 95.9%) |  |
|  | | 2019 | 95% | 87% - 98% | 96.1% (95.5% - 96.8%) |  |
|  | | 2020 | 96% | 91% - 100% | 96.8% (96.4% - 97.4%) |  |
|  | | 2021 | 96% | 91% - 100% | 97.6% (97.2% - 98.0%) |  |

^Note: A total of 117 simulations were accepted (out of 50000 simulations run).^

## Utility weighting assumptions

**Table S3 Assumed utility weighting for Quality-adjusted life year (QALY).**

| Status | Utility weight | Reference |
| --- | --- | --- |
| Not infected with HIV/using PrEP | 0.881 | [20] |
| CD4 > 350 cells/ | 0.865 | [20] |
| CD4 cell count 200-350 cells/ | 0.865 | [20] |
| AIDS stage | 0.7 | [21] |
| Living with HIV using antiretroviral drug treatment | 0.881 | [20] |

## Cost component

The costs that we included for PrEP were the local price of generic Tenofovir disoproxil fumarate/emtricitabine (TDF/FTC), the costs of visiting a physician who prescribes PrEP and the costs of monitoring side effects of tenofovir (e.g. creatinine and Urine culture) and screening sexually transmitted infections (STIs, including HIV, syphilis, hepatitis C and other bacterial STIs), according to the Dutch practice guideline [18]. We used unit prices from the year 2022 and recommended unit prices from before the year 2022 were indexed based on annual Dutch inflation as stated by the Central Bureau for Statistics. The annual costs for providing PrEP, including monitoring and costs of the drugs, are €698.19 and €43.19, if PrEP users access PrEP via the Dutch National PrEP Programme and alternative PrEP providers, respectively. More detailed information on the included costs of PrEP can be found in Table S4.

**Table S4 Annual costs per service for the use of oral pre-exposure prophylaxis (PrEP) in the Netherlands from a payer’s perspective**

| **Cost scenario** | **Type of service** | **Frequencies** | **Initial visit** | **Annual cost via the National PrEP Programme** | **Annual cost via alternative PrEP providers** |
| --- | --- | --- | --- | --- | --- |
| PrEP | HIV screening | Initial visit, 1 month after initiation, after that every 3 months | € 12,95 | € 51,80 | € 51,80 |
|  | Hepatitis C | Initial visit, after that every 12 months | € 12,65 | € 12,65 | € 12,65 |
|  | Syphilis | Initial visit, after that every 3 months | € 12,95 | € 51,80 | € 51,80 |
|  | Chlamydia, Gonorrea | Initial visit, after that every 3 months | € 70,44 | € 281,76 | € 281,76 |
|  | HBV screening | Initial visit | € 24,78 | € 0,00 | € 0,00 |
|  | Creatinine | Initial visit, 1 month after initiation, after that every 6 months | € 1,60 | € 1,60 | € 1,60 |
|  | Urine test for kidney function | Initial visit, 1 month after initiation, after that every 12 months | € 28,58 | € 28,58 | € 28,58 |
|  | Total PrEP-related tesing |  | € 176,90 | € 428,19 | € 43,19  (428,19-385)§ |
|  | PrEP | Daily intake | - | € 270,00  (€22.5/month)* | € 0,00^#^ |
| Total | | |  | € 688,19 | € 43,19 |

^Note:^

^§ There is a €385 copayment by the PrEP users for PrEP-related testing if access PrEP via alternative PrEP providers in the Netherlands per year^

^*There is a €7.50 copayment by the PrEP users for PrEP medication if access PrEP via the Nation PrEP Programme in the Netherlands^

^#There is a full cost to the PrEP users for PrEP medication if access PrEP via alternative PrEP providers in the Netherlands^

For the cost of treatments, we took the local costs by diagnosed stages into account, which included timely presenter (CD4<350 cells/mm^3), late presenter (CD4<350 cells/mm^3) and advanced presenter (CD4<200 cells/mm^3), which consist of the costs for antiretroviral drugs and direct healthcare costs, using local data [13, 15]. Direct healthcare costs included outpatient visits to an HIV specialist and to other medical specialists, hospitalization, viral load measurements, CD4 Cell count measurements, and potential additional co-medication for the late and advanced presenter [15]. The annual costs for HIV treatment, including monitoring and costs of the drugs, are €12465.92 for the timely presenter, €14541.90 for the late presenter and €24372.57 for the advanced presenter. More detailed information on the included costs related to treatment can be found in Table S4.

**Table S5 Annual costs per service for the antiretroviral drugs and other direct healthcare costs by diagnosed stage in the Netherlands from a payer’s perspective.**

| **Cost scenario** | **Type of service** | **Timely presenter** | **Late presenter** | **Advanced presenter** | **Reference** |
| --- | --- | --- | --- | --- | --- |
| Treatment component | Outpatient average visits | 567,6 | 696,6 | 761,1 | [13, 15] |
|  | Inpatient average visits | 258,02 | 1222,2 | 7115,92 | [13, 15] |
|  | Viral loads | 437,4 | 486 | 522,45 | [13, 15] |
|  | CD4 Cell count | 318,6 | 413 | 1203,6 | [13, 15] |
|  | co-medication | 0 | 839,8 | 3885,2 | [13, 15] |
|  | ART (TAF/FTC/BIC) | 10884,3 | 10884,3 | 10884,3 | [13, 15] |
| Total | | 12465,92 | 14541,90 | 24372,57 | [13, 15] |

## Figure S2. Cost-effectiveness scatterplot


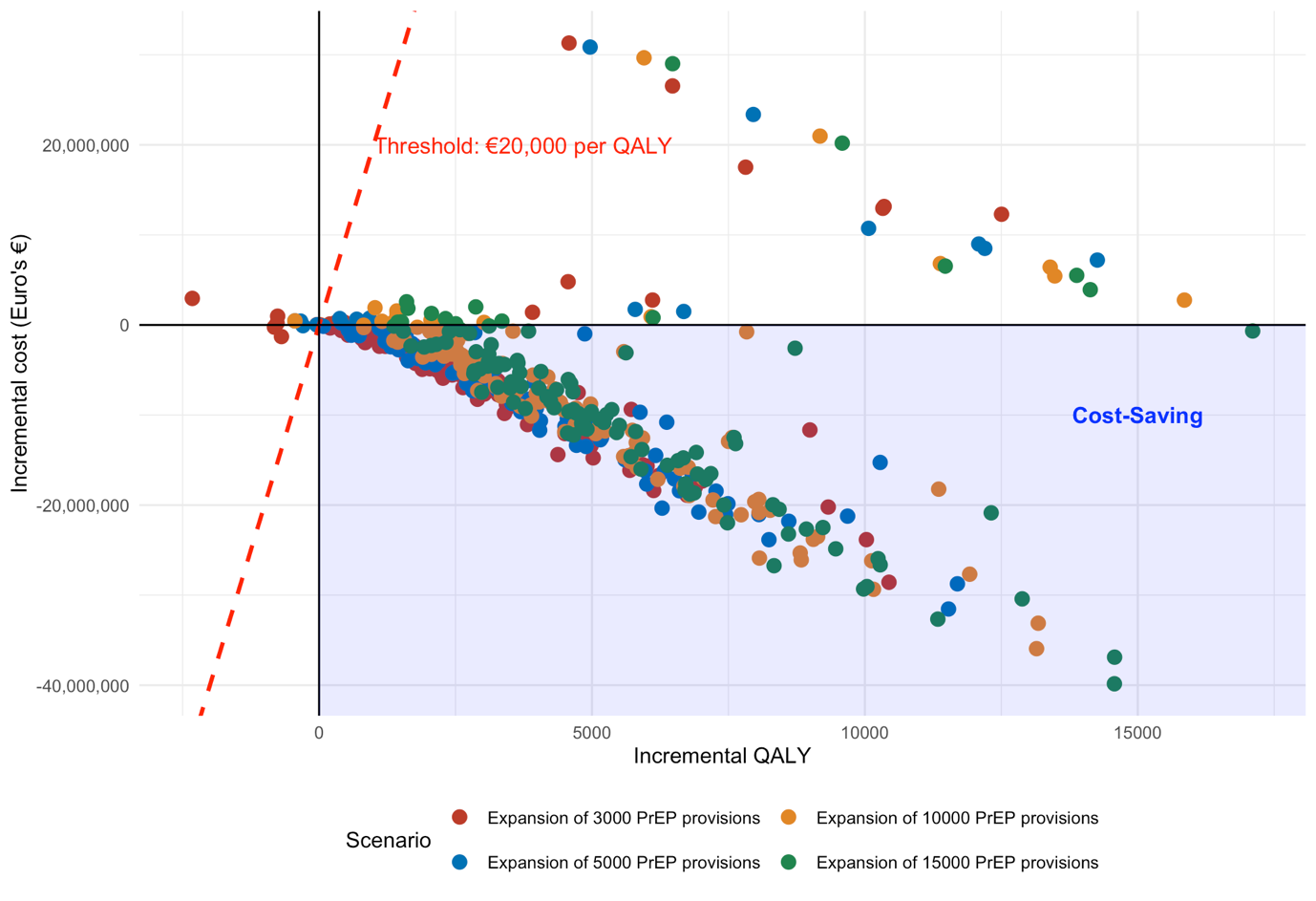


## Reference

1. van de Vijver D, Richter AK, Boucher CAB, Gunsenheimer-Bartmeyer B, Kollan C, Nichols BE, et al. Cost-effectiveness and budget effect of pre-exposure prophylaxis for HIV-1 prevention in Germany from 2018 to 2058. Euro Surveill. 2019;24(7).

2. Nichols BE, Boucher CAB, van der Valk M, Rijnders BJA, van de Vijver DAMC. Cost-effectiveness analysis of pre-exposure prophylaxis for HIV-1 prevention in the Netherlands: a mathematical modelling study. Lancet Infect Dis. 2016;16(12):1423-9.

3. Keeling MJ, Rohani P. Modeling infectious diseases in humans and animals. Princeton: Princeton University Press; 2008. 368 p.

4. Garnett GP, Anderson RM. Factors controlling the spread of HIV in heterosexual communities in developing countries: patterns of mixing between different age and sexual activity classes. Philos Trans R Soc Lond B Biol Sci. 1993;342(1300):137-59.

5. Marcus U, Hickson F, Weatherburn P, Schmidt AJ, Network E. Estimating the size of the MSM populations for 38 European countries by calculating the survey-surveillance discrepancies (SSD) between self-reported new HIV diagnoses from the European MSM internet survey (EMIS) and surveillance-reported HIV diagnoses among MSM in 2009. BMC Public Health. 2013;13:919.

6. Lodi S, Phillips A, Touloumi G, Geskus R, Meyer L, Thiebaut R, et al. Time From Human Immunodeficiency Virus Seroconversion to Reaching CD4+ Cell Count Thresholds <200, <350, and <500 Cells/mm3: Assessment of Need Following Changes in Treatment Guidelines. Clin Infect Dis. 2011;53(8):817-25.

7. Life expectancy of individuals on combination antiretroviral therapy in high-income countries: a collaborative analysis of 14 cohort studies. Lancet. 2008;372(9635):293-9.

8. Pilcher CD, Joaki G, Hoffman IF, Martinson FE, Mapanje C, Stewart PW, et al. Amplified transmission of HIV-1: comparison of HIV-1 concentrations in semen and blood during acute and chronic infection. Aids. 2007;21(13):1723-30.

9. Lodi S, Phillips A, Touloumi G, Geskus R, Meyer L, Thiébaut R, et al. Time from human immunodeficiency virus seroconversion to reaching CD4+ cell count thresholds <200, <350, and <500 Cells/mm³: assessment of need following changes in treatment guidelines. Clin Infect Dis. 2011;53(8):817-25.

10. Sood N, Wagner Z, Jaycocks A, Drabo E, Vardavas R. Test-and-treat in Los Angeles: a mathematical model of the effects of test-and-treat for the population of men who have sex with men in Los Angeles County. Clin Infect Dis. 2013;56(12):1789-96.

11. Nakagawa F, Lodwick RK, Smith CJ, Smith R, Cambiano V, Lundgren JD, et al. Projected life expectancy of people with HIV according to timing of diagnosis. Aids. 2012;26(3):335-43.

12. Jourdain H, de Gage SB, Desplas D, Dray-Spira R. Real-world effectiveness of pre-exposure prophylaxis in men at high risk of HIV infection in France: a nested case-control study. Lancet Public Health. 2022;7(6):e529-e36.

13. Nederlandse Zorg Autoriteit. TARIEVEN LABORATORIUM MEDISCHE MICROBIOLOGIE VANAF 1 januari 2022* 2022 [Available from: <https://www.scal.nl/system/files/inline/Prijzen%20website%20online%20MM%202022.pdf>.

14. Man tot man. All options to get PrEP in the Netherlands. 2023 [Available from: <https://www.mantotman.nl/en/quick-help/prep-prevents-hiv/all-options-to-get-prep-in-netherlands>.

15. Popping S, Versteegh L, Nichols BE, van de Vijver DAMC, van Sighem A, Reiss P, et al. Characteristics and short- and long-term direct medical costs among adults with timely and delayed presentation for HIV care in the Netherlands. PLOS ONE. 2023;18(2):e0280877.

16. Marcus U, Hickson F, Weatherburn P, Schmidt AJ. Estimating the size of the MSM populations for 38 European countries by calculating the survey-surveillance discrepancies (SSD) between self-reported new HIV diagnoses from the European MSM internet survey (EMIS) and surveillance-reported HIV diagnoses among MSM in 2009. BMC Public Health. 2013;13:919.

17. Wang H, Zimmermann HML, van de Vijver D, Jonas KJ. Intention and preference for long-acting injectable PrEP among MSM in the Netherlands: a diffusion of innovation approach. medRxiv. 2022:2022.11.11.22282218.

18. Bierman W, Hoornenborg E, Nellen J. Nederlandse multidisciplinaire richtlijn Pre-expositie profylaxe (PrEP) ter preventie van hiv (update 2022) 2022 [Available from: <https://www.soaaids.nl/files/2022-07/20220711-PrEP-richtlijn-Nederland-versie-3-update-2022.pdf>.

19. van Sighem AI, Wit F, Boyd A, Smit C, Matser A, Reiss P. Monitoring Report 2023. Human Immunodeficiency Virus (HIV) Infection in the Netherlands. Amsterdam: Stichting HIV Monitoring, 2022. 2023 [Available from: <https://www.hiv-monitoring.nl/en/resources/monitoring-reports>.

20. Popping S, Kall M, Nichols BE, Stempher E, Versteegh L, van de Vijver D, et al. Quality of life among people living with HIV in England and the Netherlands: a population-based study. Lancet Reg Health Eur. 2021;8:100177.

21. Tengs TO, Lin TH. A meta-analysis of utility estimates for HIV/AIDS. Med Decis Making. 2002;22(6):475-81.
